# Evaluating the organisational climate in Italian public healthcare institutions by means of a questionnaire

**DOI:** 10.1186/1472-6963-7-73

**Published:** 2007-05-22

**Authors:** Ulrich Wienand, Renata Cinotti, Augusta Nicoli, Miriam Bisagni

**Affiliations:** 1 Azienda Ospedaliero Universitaria di Ferrara "Arcispedale S. Anna", corso Giovecca 203, 44100 Ferrara, Italy; 2 Agenzia Sanitaria Regionale Emilia – Romagna, viale Aldo Moro 21 – 40127 Bologna, Italy; 3 Azienda Usl di Piacenza, via Taverna 49, 29100 Piacenza, Italy

## Abstract

**Background:**

By means of the ICONAS project, the Healthcare Agency of an Italian Region developed, and used a standardised questionnaire to quantify the organisational climate. The aims of the project were (a) to investigate whether the healthcare institutions were interested in measuring climate, (b) to estimate the range of applicability and reliability of the instrument, (c) to analyse the dimensions of climate among healthcare personnel, (d) to assess the differences among employees with different contractual positions.

**Methods:**

The anonymous questionnaire containing 50 items, each with a scale from 1 to 10, was offered to the healthcare organisations, to be compiled during ad hoc meetings. The data were sent to the central project coordinator. The differences between highly specialised staff (mostly physicians) and other employees were assessed after descriptive statistical analysis of the single items. Both Principal Component Analysis and Factor Analysis were used.

**Results:**

Ten healthcare organisations agreed to partecipate. The questionnaire was completed by 8691 employees out of 13202. The mean value of organisational climate was 4.79 (range 1–10). There were significant differences among single items and between the 2 groups of employees. Multivariate methods showed: (a) one principal component explained > 40% of the variance, (b) 7 factors summarised the data.

**Conclusion:**

Italian healthcare institutions are interested in assessing organisational phenomena, especially after the reforms of the nineties. The instrument was found to be applicable and suitable for measuring organisational climate. Administration of the questionnaire leads to an acceptable response rate. Climate can be discribed by means of 7 underlying dimensions.

## Background

The Italian Healthcare Service has undergone major restructuring since new laws passed in 1992 and 1993. These laws provided a transition from a predominantly public system with funding unrelated to the number and specific type of services delivered or to performance, to a public-private mix that includes market elements, managed competition, and a kind of "financing by output" system.

In order to function efficiently Italian healthcare organisations, must have reliable management tools. Economic and analytic accounting systems have been introduced based on cost centres, and budgets developed from strategic goals negotiated between the management board and the units. These management tools allow the maintainance of an attainable equilibrium between results and resources. This is a radical innovation, especially from physicians' point of view, as they previously considered resources unlimited.

There is an increasing use of specific techniques to evaluate employees' individual performance, and the Italian law permits a considerable percentage of doctors' and nurses' renumeration to be linked to the attainment of clinical and economical goals.

There have also been important changes for the nursing professions: their education moved from hospital-owned schools to the medical faculties of the universities, and in many hospitals the chain of command of nurses has been separated from that of doctors. There are also considerable problems in recruiting new nursing staff.

All this upheaval has resulted in the loss of reference points for organisations, managers and staff. The context has changed quickly, but the people and the organisations were not prepared for this. Recently several Italian healthcare organisations have started to analyse the "organisational climate", instituting a feedback channel to determine what kind of support would best help staff adapt to, exploit and thrive in this new climate. A survey on organisational climate is one method that can be used to monitor and improve employees' involvement in the changes.

## Methods

In 1999 the Regional Healthcare Agency of Emilia-Romagna promoted a region-wide CQI (Continuous Quality Improvement) program. Within this framework an expert group of psychologists and sociologists developed a specific instrument to measure organisational climate that could be used by all healthcare organisations in the region with the following criteria:

• provide a tool for communication between management and employees;

• allow self assessment of healthcare organisations in a CQI perspective;

• stimulate and improve the involvement of employees during the transition and subsequently.

Members of the expert group came from 8 different healthcare organisations of the region; all of them had previous experience with measuring employee satisfaction and motivation, organisational climate or value-systems.

A search of the literature about previous experiences in healthcare organisations was performed. On the basis of the results of the search, 150 items were formulated. Applying the "Nominal Group Technique" (NGT) the team reduced the number of items to 50. The formulation of items followed some simple rules: clear language, no double negation, wording that hopefully would elicit the respondent's spontaneous reaction. Every item contains only a single concept in order to obtain a univocal response. The responses to the 50 items are graduated on a "self-anchoring" scale; the value assigned by the subject is one on a scale from 1 to 10, where the subjective distance between any two consecutive values is assumed to be equal. No semantic correspondences were attributed to the values.

The anonymous self-administered questionnaire was made up of 4 sections:

0. Instructions

1. Generic items,

2. Items related to the entire healthcare organisation,

3. Items related to the employee's ward, service, or unit.

Within each of the sections 1 to 3, items follow a randomised sequence in order to avoid bias due to phenomena like "response set". No personal information was recorded.

The first version of the questionnaire was tested in 1999 in a small hospital in the region with 173 employees. In this pilot study, the interviewees were asked to complete the questionnaire, and comment on it. The questionnaire was found to be easy to fill in and comprehensible. The employees emphasised the importance of anonymity.

The project entitled Survey on Organisational Climate in Healthcare Institutions (in Italian 'Indagine Clima Organizzativo Nelle Aziende Sanitarie') was given the acronym ICONAS.

The aims of the ICONAS project were:

• Assess the interest in using the instrument in healthcare organisations

• Evaluate the large-scale applicability of the instrument

• Check the internal consistency and the reliability of the questionnaire

• Analyse the principal dimensions of the organisational climate

• Identify possible significant differences among various groups of employees.

The Regional Health Agency then created the ICONAS package made up of the questionnaire, validation by means of NGT and pretest, the instructions for its administration and a data base in "Access^®^" for data entry. The package was made available free of charge, the only requirements were that the authors and the promoting Healthcare Agency were to be explicitly cited and that a copy of the data base was to be transmitted to the project coordinator

This paper summarises the results of the project.

### The population surveyed

Ten public-sector health care institutions accepted the invitation to participate in the project: 2 hospital trusts and 8 local healthcare units in various parts of Northern Italy. Local healthcare units are organisations that include both hospitals and out patient clinics.

Some of the institutions were within the Emilia-Romagna region, whose agency had promoted the project, but other institutions came from other Italian Regions.

Employees in various organisational environments took part in the survey which could cover:

• the entire healthcare organisation,

• one or more departments,

• one or more units (wards, clinics, diagnostic services).

"Department" is an autonomus suborganisation of the entire organisation and is made up of several units.

A total of 13,202 employees were invited to participate in the survey on organisational climate, regardless of tenure. It was sufficient that the employees of the organisation itself were present at a particular date. University post-graduate students and employees of external collaborating firms were excluded.

The survey was performed during the period 2000 – 2004. On average, the time needed for preliminary communication, administration of the questionnaire, data analysis, presentation of the results and transmission of the data to the project coordinator was 3–4 months for each of the 10 sites.

The national contracts for healthcare workers were used to define categories of staff. Physicians, other scientists and management were placed in the first category and are referred to here as highly specialised staff. The second categaory used was specialised and unspecialised employees. About 78% of the employees belong to the second category. About 80% of staff classified as "highly specialised" were physicians, whereas the majority of "specialised and unspecialised employees" were nurses (including head nurses), therapists, and laboratory and radiology technicians. This classification was not changed during the survey, and is still valid. One organisation did not distinguish correctly between the 2 contractual positions, and included nurses and rehabilitation therapists in the "highly specialised" category. For this organisation, this variable had to be recoded as missing.

### Administration of the questionnaire

The preferred way of administering the questionnaire was that suggested by the regional expert group :

• The organization's management publicly supported the study; the CEO sent a personal letter to each employee, explaining the aims of the survey, requesting collaboration and guaranteeing anonymity.

• High- and intermediate-level managers were personally involved.

• Training meetings were held with the staff chosen to illustrate the questionnaire, the method of administration and the collection of the completed forms.

• These persons then held ad hoc meetings with their target groups to administer the questionnaire.

• After data analysis further meetings were held to present the results.

• Results were transmitted to the ICONAS project coordinator.

The questionnaires were completed by the employees during the ad hoc meetings described above and were personally dropped through a slit into an official box to further ensure anonymity. The meetings during which the questionnaire was to be filled out were considered as paid working time. The average amount of time spent filling out the questionnaire was 20 minutes.

The number of meetings required ranged from 4 to 40 depending on the number of employees to contact, their location, shift working, the number of employees to be recruited during each session.

### Ethics

The study is neither a biomedical experimentation nor a clinical trial, but an organisational survey. The subjects involved were not medical patients but employees of the partecipant healthcare institutions. This kind of research is not within the jurisdiction of Healthcare Ethics Committees according to Italian law.

The institutions' management publicly supported the study and formally approved the methodology. The employees and the trade unions were informed of the right of any subject to abstain from partecipation to the study. A number of employees did claim this right (see "Results").

On the cover sheet of the questionnaire was a written statement guarenteeing the anonymity for each subject and assuring that data processing was only on an aggregate level.

### Data analysis

An empty database in "Microsoft Access^®^" was prepared and distributed to all the participating institutions for data entry and preliminary analysis. In order to save time, outsourcing the data entry and/or analysis was permitted, as an alternative to data entry by specially designated employees. The questionnaire was to be administrated without alteration and a copy of the database was to be returned to the ICONAS project coordinator within a specified time.

Descriptive statistics (quantiles, mean values, measures of dispersion, modes) were progammed into the database. Kruskall-Wallis analysis of variance, principal component analysis and factor analysis were combined to provide a framework for the interpretation and presentation of the pooled results. The statistical packages used by the regional working group were SPSS^® ^(for Windows, version 11.5) and SAS^® ^to confirm the multivariate analyses. We checked the normality of the distributions by means of histograms, descriptive statistics, including skewness and kurtosis, and the Kolmogorov-Smirnoff test.

## Results

### Response rates

8681 of the 13202 employees who had been invited to respond to the questionnaire (66%) did so (see Table 1). The percentage of responders ranged from 60% to 97% in the 9 institutions that used the suggested method of administering the questionnaire. One organisation sent the questionnaire by mail to the employees, with recall after 2 weeks, and had the lowest response rate, 44%.

**Table 1:** Organisations that used the ICONAS questionnaire

**Code**	**Type of Organisation**	**Population**	**No, employees contacted**	**No, responding**	**% response**
5	Local Healthcare Unit	Whole institution	4,690	2,951	62.9%
6	Local Healthcare Unit	Whole institution	1,853	1,589	85.8%
2	Hospital Trust	Whole institution	2,447	1,582	64.7%
10	Local Healthcare Unit	Whole institution	2,300	1,012	44.0%
4	Local Healthcare Unit	Department of Longterm and Home Care for Elderly	306	227	74.2%
3	Hospital Trust	Department of Obsthetrics and Paediatrics	300	181	60.3%
7	Local Healthcare Unit	Department of Public Health	116	113	97.4%
8	Local Healthcare Unit	25 single units	900	799	88.8%
9	Local Healthcare Unit	3 single units	181	155	85.6%
1	Local Healthcare Unit	1 single unit	109	72	66.1%

	**Total**		**13,202**	**8,681**	66.0%

Questionnaires were totally completed by 6890 employees (79.4% of the responders), and completely blank questionnaires were returned by only 5 persons. Response to each of the 50 individual items ranged from 95% to 99%. The item about "adequate management of internal conflicts within the unit" had the highest nonresponse rate, i.e. 5%.

8372 employees (96%) completed the item about level of specialisation: 1835 (22%) were highly specialised and the remaining 6537 (78%) were specialised and unspecialised.

Response was best for Part 1 of the questionnaire (92.7%), followed by Part 2 (88.5%).

When the response rates to the single items of each part of the questionnaire were considered, the largest percentage of nonresponse was in the third part about decision-making and internal relationships within the unit.

### Use of the scale

**Figure 1:**
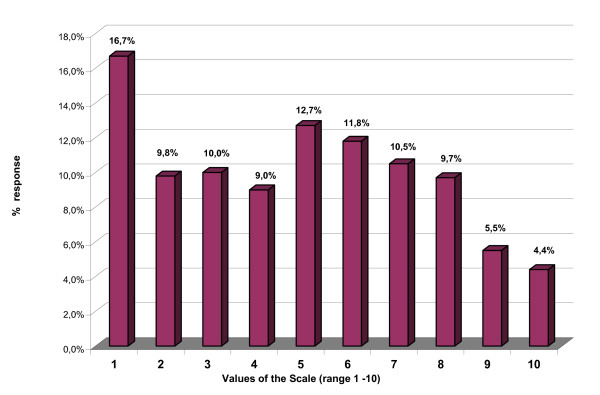
**Answers to the individual items**. The items have a bimodal distribution.

Every value of the self-anchoring scale of possible responses, ranging from 1 to 10 was used at least once. The median response for all items was 5 (58.2% of the values were below this), quite near the midrange of 5.5. The distribution was bimodal, with one mode at the value 1 and a less accentuated one at the value 5 (Figure 1).

**Figure 2:**
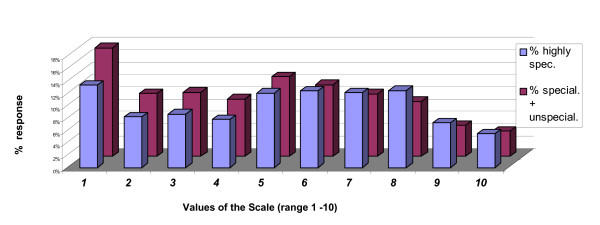
**Answers to the items by contractual position**. The answers to the items by contractual position (highly specialised vs specialised and unspecialised) have both a bimodal distribution.

For each of 2 categories of specialisation the principal mode was at 1; for the "highly specialised employees" the secondary mode was at 8, whereas for the "specialised and unspecialised employees" the secondary mode was 5 (Figure 2).

### Descriptive statistics for organisational climate

The mean value for organisational climate was 4.79 (s.d. = 1.55, s.e. = 0.017, CV = 0.322). The first quartile was 3.66, the median 4.78 and the third 5.88. The median was 4.78, almost identical to the mean, reflecting the lack of skewness.

The values of the coefficient of variation (CV) were similar in the various organisations, being more similar (CV 0.30 to 0.34) in the organisations that involved all employees, and spreading wider (CV 0.26 to 0.38) in those organisations that selected certain departments or units.

**Figure 3:**
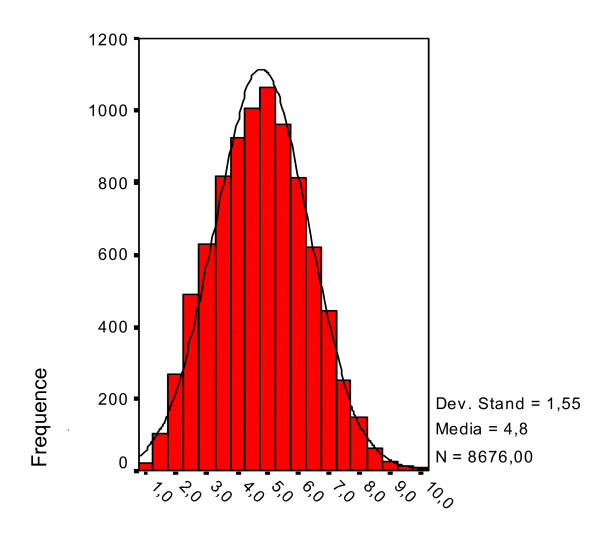
**Distribution of the overall questionnaire means**. Stand. Dev. = 1.55. Means = 48. N = 8,676

A preliminary analysis of the pooled data: means, standard deviation, skewness and kurtosis, (respectively 0.142 and -0.366), indicated a trend towards platikurtosis; this was confirmed by the Kolmogorov-Smirnov test (p = 0.0001). The histogram in Figure 3 shows a slight deviation from the Gaussian curve (Figure 3).

Considered individually, the distributions and mean values of some of the 50 items differ markedly from those of the overall data. The mean values range from 2.9 for item #20 ("knowledge of the organisation's action plan") to 7.3 for item #37 ("concern for the patients' requests shown by the colleagues"), as well as for item #5 ("feeling of responsability during daily work").

**Figure 4:**
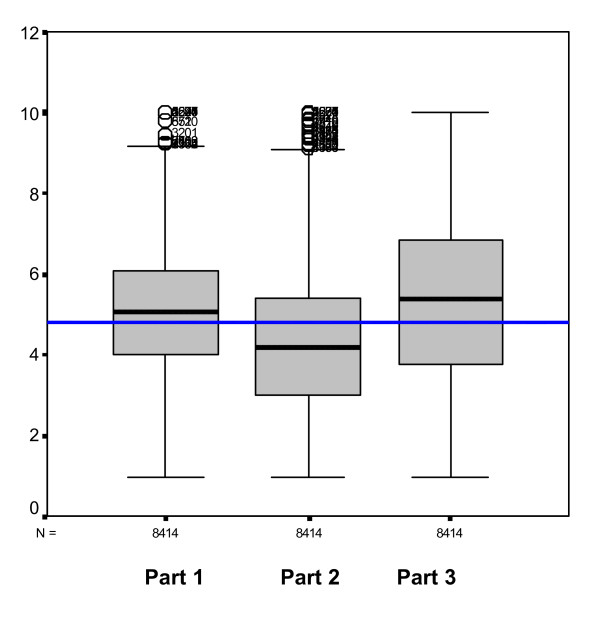
Box plots of the means of the 3 parts of the questionnaire.

Part 3 had the highest mean, 5.3, followed by Part 1, 5.0, and Part 2, 4.27, see Figure 4. The differences among these 3 overall means of the 50 variables we considered are significant (2-tailed Kolmogorov-Smirnov test P = 0,001)

The perceived organisational climate has higher values in the third part of the questionnaire, regarding internal processes in the unit, that are closer to the employees, while the organisation-wide processes and changes have lower values, but also a considerable number of outliers towards the top of the scale.

**Figure 5:**
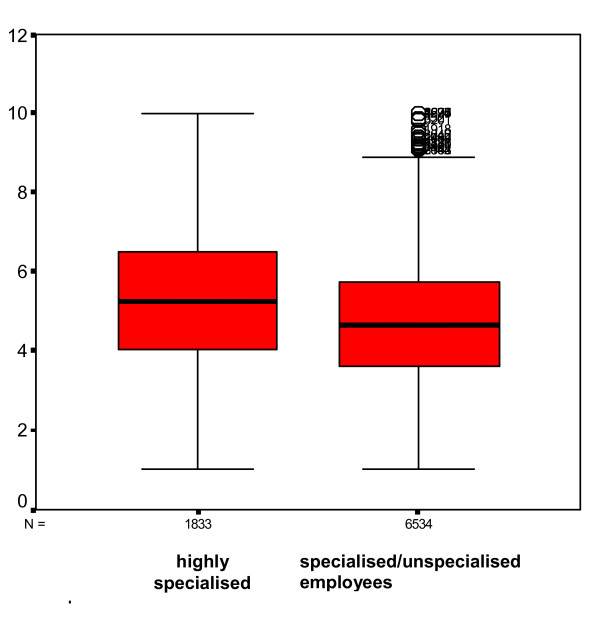
**Box plots of the overall questionnaire means**. Means for highly specialised staff vs specialised/unspecialised employees

The difference in overall means of the variables on organisational climate for the highly specialised staff (mean 5.2, sd 1.6) vs. specialised and unspecialised staff (mean 4.6, sd 1.4) was highly significant (2-tailed Kruskall-Wallis test P =< 0.001) (Figure 5).

The perceived organisational climate has higher values among highly specialised employees (e.g. mainly doctors), while among specialised (e.g. nurses) or unspecialised employees the mean value is lower, but there are many outliers towards the top of the scale.

The same was true for 47 of the 50 single items. The 3 nonsignificant items were #2: "usually consulting colleagues in unexpected situations", asymptotic p = 0.100; #7: "on the job teamwork", asymptotic p = 0.207; #13: "sense of security as a result of the reorganisation", asymptotic p = 0.631, with p > 0.05. The only variable with a higher mean for the specialised and unspecialised employees (5.33) with respect to highly specialised staff (5.16) involved the continuous education programmes offered and promoted by the organisation.

The largest value of standard deviation, 2.9, was found for item #33 about the "consistency between the incentive system and the organisation's goals", while a standard deviation of 2.0 was found for item #18 about the "efficacy of internal communication among the various sectors of the organisation".

### Summarising the data

We used orthogonal principal component analysis (PCA), to document the fundamental structure underlying the variability of the data about organisational climate. PCA transformed the set of observations into a simpler structure which was almost as informative as the original data. This was confirmed by the Kaiser-Meyer-Olkin (KMO) sample adequacy test value of 0.975 and the Bartlett sphericity test, p = 0.001. We extracted the same number of components as there were variables (50). The validity of the extraction is determined by the percentage of variance, at least between 60% and 80%, that the principal components explain.

The variance explained by the 7 principal components with initial eigenvalues greater than 1 is 62.3%. Missing data was treated listwise, i.e. only subjects with complete data for the variables in the analysis were considered.

An important component explained 40% of the variance by itself, and was interpreted as organisational climate of the institution. This component was considered to be the confirmation of the content validity of the items: all tended to characterise the same phenomenon and there were no items that could be considered extraneous to the content.

Table 2 reports the weight of each variable in the component. This analysis was a further step in the validation process.

**Table 2:** Principal component number 1

24: recognition of good results by the organisation	0.77
17: concern of the General Management about the employees' needs	0.76
30: clarity in the way the General Management evaluates performance	0.75
34: respect for enterprisingness within the organisation	0.75
49: support for improvement of competence	0.75
46: clear aims stated by the unit's chief	0.74
39: consideration of the employees' opinions by the director of the unit when decisions are made	0.73
41:clarity of the incentive system in the unit	0.73
47: participation in decisions undertaken within the unit	0.73
18: efficacy of the General Management's system of internal communication among the various sectors of the organisation	0.72
19: propensity to advise others to work in the organisation	0.71
21: clarity in the assignment of job specifications by General Management	0.71
25: aims clearly defined by the General Management	0.71
45: propensity to advise others to work in the unit	0.71
50: encouragement by the unit's chief to improve professional knowledge	0.71
23: being proud to work in the organisation	0.70
33: consistency between the incentive system and the organisation's goals	0.70
44: comprehensibility of projects and aims of the unit	0.70
31: quality of services provided by the organisation	0.69
42: adequate management of internal conflicts within the unit	0.69
10: increased motivation after the transformation of the organisation into a public company	0.67
40: efficacious flow of information within the unit	0.67
13: sense of security as a result of the reorganisation	0.66
43: continuous education programs offered and promoted within the unit	0.66
16: clarity of the organisation's incentive system	0.65
28: efficacious flow of information within the organisation	0.64
29: propensity to teamwork within the organisation	0.64
6: personal involvement in decision processes which could affect one's job	0.64
8: propensity of units to collaborate with each other	0.63
1: recognition of one's on-the-job daily achievements	0.62
12: job satisfaction	0.62
7: on the job teamwork	0.62
27: continuous education programs offered and promoted by the organisation	0.61
20: knowledge of the organisation's action plan	0.59
32: possibility of decision making for the chiefs of the units	0.59
36: propensity to teamwork within the unit	0.59
4: ability of the system to reward individual performance	0.59
35: simplicity of roles and assignment of tasks within the unit	0.58
26: concern of the General Management about customers' complaints	0.57
14: equitable distribution of wages	0.54
22: concern of colleagues in the organisation about patients' needs	0.51
11: knowledge of the department's structure	0.49
15: knowledge of the organisation's mission and vision	0.49
48: adequacy of technical equipment	0.49
3: knowledge of the organisational structure of the General Management	0.48
37: concern for the patients' requests shown by the colleagues in the unit	0.46
38: adequate working conditions in the unit	0.44
5: feeling of responsability during daily work	0.43
9: feeling of self-direction in daily work situations	0.42
2: usually consulting colleagues in unexpected situations	0.30

The analysis of the 50 variables was refined to include the synthetic dimensions that characterised the phenomenon of organisational climate, so as to facilitate the use of the data. Maximum-likelihood Factor Analysis, with varimax rotation of the orthogonal axes and Kaiser normalisation was then performed. A KMO value of 0.975 was evidence of the adequacy of the sample, explained variance was 62.3% of the total variance, and the number of factors extracted (7 with eigenvalues > 0.3) were consistent with the results of the principal component analysis (Table 3).

**Table 3:** The 7 main factors

**Factor**	**Loading**	**Explained variance (%)**	**Number of items**	**Crohnbach's Alpha**	**Mean**
1: Performance assessment and rewarding systems	9.70	17	16	0.945	3.68
2: Leadership style in the unit	8.44	15.8	13	0.945	5.16
3: Job satisfaction	3.19	7.5	6	0.831	6.08
4: Organisational communication	2.68	5.3	4	0.842	4.06
5: Perceived quality of care	2.37	4.9	5	0.815	5.24
6: Team spirit	1.47	3.2	3	0.760	6.15
7: Training and development	1.50	2.3	3	0.764	5.14

Validity and internal consistency between items on the scale of each factor were checked by reliability analysis and Cronbach's α. The values of Cronbach's α, above 0.6 for each factor, confirm the validity and internal consistency for each of the considered scales.

Mean values were then recalculated with the values of this analysis for each of the 7 factors.

**Figure 6:**
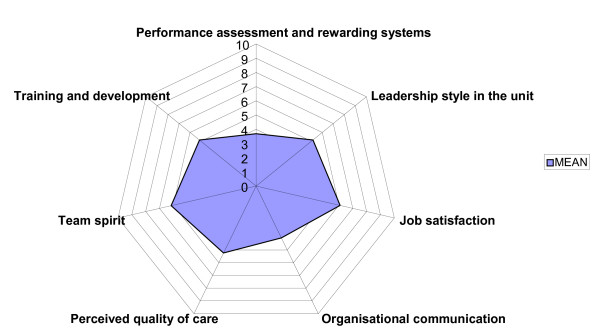
Organisational climate – the 7 main factors.

Pooling the items on the considered scales resulted in the exclusion of 2 items, #2 and #38, respectively about consulting colleagues in unexpected situations and the adequate conditions of the workplace (see Table 4 and Figure 6)

**Table 4:** Factor analysis – rotated factor matrix^i^

**Item**	Performance assessment and rewarding systems	Leadership style in the unit	Job satisfaction	Organisational communication	Perceived quality of care	Team spirit	Training and development
	
	**1**	**2**	**3**	**4**	**5**	**6**	**7**
17: concern of the General Management about the employees' needs	**0.75**						
16: clarity of the organization's incentive system	**0.69**						
33: consistency between the incentive system and the organisation's goals	**0.69**						
18: efficacy of the General Management's system of internal communication among the various sectors of the organisation	**0.66**						
24: recognition of good results by the organisation	**0.66**	0.31			0.30		
4: ability of the system to reward individual performance	**0.66**						
10: increased motivation after the transformation of the organisation into a public company	**0.63**		0.37				
13: sense of security as a result of the reorganisation	**0.61**		0.39				
21: clarity in the assignment of job specifications by General Management	**0.61**						
30: clarity in the way the General Management evaluates performance	**0.60**				0.34		
34: respect for enterprisingness within the organisation	**0.60**				0.31		
25: aims clearly defined by the General Management	**0.59**						
14: equitable distribution of wages	**0.54**						
19: propensity to advise others to work in the organisation	**0.50**		0.36		0.41		
28: efficacious flow of information within the organisation	**0.49**						**0.33**
8: propensity of units to collaborate with each other	**0.42**		0.35				
46: clear aims stated by the unit's chief		**0.78**					
50: encouragement by the unit's chief to improve professional knowledge		**0.77**					
47: participation in decisions undertaken within the unit		**0.74**					
49: support for improvement of competence		**0.73**	0.31				
42: adequate management of internal conflicts within the unit		**0.73**					
39: consideration of the employees' opinions by the director of the unit when decisions are made	0.31	**0.68**					
45: propensity to advise others to work in the unit		**0.67**	0.35				
44: comprehensibility of projects and aims of the unit		**0.65**					
43: continuous education programs offered and promoted within the unit		**0.62**					**0.52**
40: efficacious flow of information within the unit		**0.60**					
41:clarity of the incentive system in the unit	0.50	**0.57**					
35: simplicity of roles and assignment of tasks within the unit		**0.51**				0.37	
48: adequacy of technical equipment		**0.38**					
12: job satisfaction			**0.65**				
1: recognition of one's on-the-job daily achievements		0.34	**0.55**				
7: on the job teamwork			**0.53**				
6: personal involvement in decision processes which could affect one's job		0.36	**0.53**				
5: feeling of responsability during daily work			**0.47**				
9: feeling of self-direction in daily work situations			**0.46**				
3: knowledge of the organisational structure of the General Management				**0.77**			
11: knowledge of the department's structure				**0.75**			
15: knowledge of the organisation's mission and vision	0.33			**0.63**			
20: knowledge of the organisation's action plan	0.46			**0.54**			
31: quality of services provided by the organisation	0.41				**0.55**		
23: being proud to work in the organisation	0.41		0.44		**0.49**		
22: concern of colleagues in the organisation about patients' needs					**0.48**		
26: concern of the General Management about customers' complaints	0.39				**0.44**		
32: possibility of decision making for the chiefs of the units	0.38				**0.41**		
36: propensity to teamwork within the unit		0.51				**0.55**	
37: concern for the patients' requests shown by the colleagues in the unit		0.36				**0.49**	
29: propensity to teamwork within the organisation	0.30					**0.43**	
27: continuous education programs offered and promoted by the organisation	0.34						**0.66**
*38: adeguate working conditions in the unit*							
*2: usually consulting colleagues in unexpected situations*							

^i ^Maximum lilelihood extraction – Varimax rotation with Kaiser normalisation

## Discussion

The ICONAS questionnaire was administered to 8681 employees with various professional profiles in the Italian Healthcare System, and this makes it the most widely used instrument to measure organizational climate in this setting. We did not find previous comparable experiences in Italy in the literature. Even on the international scene, few specific attempts to measure organisational climate in healthcare institutions involving all the professionals present in the organisation and emphasising a global measure of organisational climate have been made [1-3]. More often, measurement of organisational climate has been attempted in different environments, and regarding various professions, to compare instruments, topics and concepts [4-10].

The international studies mainly measure variables pertaining to job satisfaction [11-15], in specific sectors of the healthcare field or for particular groups of professionals [16-19]. The lack of a uniform terminology regarding organisational climate affects the measurement instruments used in the healthcare field [20]. Hale [21] pointed out various problems encountered in measuring job satisfaction, and criticised the multiplicity of the instruments proposed, suggesting that the instruments used to measure job satisfaction across different types of settings are often neither valid nor reliable. The scales used to measure job satisfaction are often drawn up for specific purposes such as to quantify absenteeism, the mobility of nurses, or the impact of a change in the clinical governance system [22].

Specific studies to investigate the application of an instrument to measure organisational climate are not less problematic, and are also less frequent.

Recently, Gershon [22,23] published a systematic review of the measurement of organisational climate, enumerating the properties that these instruments should have to be useful and amenable to standardisation. During the last 5 years, studies of this type have been used in the field of healthcare; at the same time interest in the search for evidence of validity and reliability has grown. Periodic assessment of the organisational climate, "taking the pulse of the organisation", plays an important role in the eyes of administrators and managers, when considered together with the results of quality assessment in a healthcare unit [22,24].

The ICONAS questionnaire on organisational climate was offered to a total of 13,202 employees in various Italian healthcare institutions in the public sector which are involved in processes of organisational change and quality improvement. In the Emilia-Romagna region 6 organisations adhered to the survey, as did 4 more institutions in 3 other regions with different models of healthcare policy.

The method of administrating the questionnaire in ad hoc meetings, with completed forms collected at the end of the session, helped to obtain a good response rate. This rate was similar to those obtained in other studies to measure climate, staff perception or job satisfaction in healthcare organisations [1,12,19,22,25-28]. It was not possible to take into account the method of administration when comparing response rate, as this was not always reported in the other studies.

The ICONAS project coordinator maintained contact with and explicitly requested feedback from the organisations that used the questionnaire. The fact that there were no complaints about the understandability, the number of items or the response scale confirms the validity of the results about the structure of the questionnaire pre-tested in 1999 in a small hospital.

Hale [21] emphasised the importance of the assessment of measurement instruments designed to identify the underlying dimensions of the phenomenon under investigation, and this led us to analyse in detail the choice of the methodologies to apply and to specify in detail the contexts in which each instrument was used. In contrast, many studies used Principal Component Analysis (PCA) and Factor Analysis (FA) to identify the dimensions underlying job satisfaction or organisational climate, but the reasons for the choice were not described.

PCA is an orthogonal linear transformation that transforms the data such that the greatest variance is extracted from the data (called the first principal component). It then removes this variance and finds a second linear combination that explains the maximum proportion of the remaining variance, and so on. PCA can be used to reduce the dimensionality of a data set by retaining those characteristics of the data set that contribute most to its variance. The reduction on dimensionality is accomplished by keeping lower-order principal components and ignoring higher-order ones.

On the other hand, FA is a statistical data reduction technique used to explain variability among observed random variables in terms of fewer unobserved random variables called factors. The observed variables are expressed as linear combinations of the factors plus random terms.

These two approaches (PCA and FA) appear to be similar since both are used to reduce a large set of variables to a much smaller number of dimensions or underlying factors. However, important differences between PCA and FA are often overlooked. PCA is particularly indicated in situations in which the best possible empirical combination is sought, namely the combination that explains the largest proportion of the variability in the initial correlation matrix of all the variables. Instead, FA should be used to obtain a hypothetical solution or to confirm a hypothesis. Several techniques can be used to extract factors that describe dimensions underlying the variables and their relationships. Caution should be used when rotating the factors, which should only be done if it helps to interpret the underlying factors.

In this study, we checked the validity of the items in the questionnaire and the fundamental dimensions of the phenomenon being investigated with both approaches.

In the original formulation of the questionnaire constructed by means of the NGT technique, we had conjectured that the items would cover most of the variability of the phenomenon known as organisational climate and agreed upon by the group of experts as "the collective representation of the quality of the internal relationships within a structured set of people", with reference to Schneider's reflections on organisational change and the weak coupling described by Weick [21,29].

By means of PCA without rotation applied to the 50 variables of the ICONAS questionnaire, we confirmed the validity of the items in the questionnaire and the minimal organisational level involved in order to improve the organisational climate.

The first component explained 40% of the variance, had an eigenvalue > 20 and contained all 50 items of the questionnaire, confirming the hypothesis that all 50 items were necessary to explain the single phenomenon organisational climate, whose factor loadings ranged from 0.77 to 0.30.

The second component, the organisational archipelago, which explained 7% of the variance and had an eigenvalue >3, showed that the organisational structure within a healthcare institution that influences the organisational climate most is the unit. The climate of the unit is experienced as a happy island in an organisational archipelago whereas the latter tends to have a critical, problematic and extremely variegated climate. The archipelago exists even when there is no interchange among the islands, each of which is in this case really an island, self-sufficient and self-contained. This metaphor describes the characteristic of an weakly linked organisation called loose coupling [29,29], which applies to an organisation in which the events and the parts of the organisation that are linked are open to reciprocal influence, but each preserves its own identity and its own separateness, both physical and otherwise. There is something which connects them; they are bound to each other, but this bonding can be weak, limited to a few aspects, fragile and weak. A loosely coupled organisation is able to adapt to the complexity of the environmental variables and the polymorphous structure of healthcare organisations.

FA with varimax rotation explained 62.3% of the variance and led to the extraction of the factors underlying the phenomenon referred to as climate. We found 7 factors, which we interpreted in order of factor loadings:

1. Performance assessment and reward systems

2. Leadership style in the unit

3. Job satisfaction

4. Organisational communication

5. Perceived quality of care

6. Team spirit

7. Training and development.

The reliability of the construct of each factor was confirmed by values of Cronbach's α between 0.945 and 0.764 for each of the 7 factors.

These factors should underlie the strategies used to implement changes under the hypothesis that measuring climate periodically will be useful in monitoring the changes in the organisation and an indicator of perceived internal quality.

The highest mean values corresponded to the factors Team spirit and Job satisfaction. These dimensions are reported by others to be among the most important. The employees in the Italian healthcare organisations we studied gave the highest scores precisely to these central aspects of subjective experience.

The aspects that pertain to the cognitive aspects of the new organisational setting (with respect to the action plan, mission, vision, rewarding system) had the lowest scores. The perceived attention paid to the needs of the professionals was also rather low. The items pertaining to the smallest and nearest milieu, the employee's unit, were perceived more positively than those referring to the whole organisation. The question most often left blank was the question about the management of conflicts.

The most important factor, and at the same time the most critical one, grouped the items about performance assessment and reward systems. It can be conjectured that the introduction of principles of efficiency into the Italian healthcare system was experienced more negatively than the other dimensions that make up climate.

Highly specialised staff responded more positively about almost all the items. Nurses, technicians and other non highly specialised staff were more positive about professional training. Both groups gave similar answers for the variables pertaining to team spirit.

Severinsson e Hummelvoll [30], using a questionnaire on job satisfaction of nurses and the work environment in acute psychiatric care, extracted 5 factors labelled stress and experiences of shortcomings, general satisfaction, managerial support, communication and cooperation and professional development [30]. Chou, Boldy and Lee developed a 5-factor model of staff satisfaction: personal satisfaction, workload, team spirit, training and professional support. Of these, the lowest level of satisfaction overall applied to workload and the highest to team spirit. [31]

The 7 dimensions we extracted show a different factorial structure but correspond with these authors for the dimensions job satisfaction, team spirit and training and development.

Tovey and Adams explored the sources of nurses' job satisfaction in the 1990s. "New sources of satisfaction and dissatisfaction emerged, directly associated with change arising out of the NHS internal market. These include pressures associated with new roles, role conflict, lack of job security, 'tight resources', using new technology, a perceived lowering of standards of patient care, coping with increased amounts of paperwork, and the experience of working in a rapidly and constantly changing environment" [32]. Also Arnetz reports evidence of a relationship between organizational changes and lower job satisfaction. Our study seems to indicate that profound organisational changes have strongly conditioned organisational climate.

## Conclusion

Organisational changes are possible only if the persons within the organisation change; if they do not, the organisation will not. Schneider states "here is the central point: organizations as we know them are the people in them; if the people do not change, there is no organizational change"[33], and recognises the nature of the interpersonal relations among the members of the organisation to be an important dimension of climate.

It is also possible to document cultural aspects of the organisation and the changes that take place [34], but it is easier to find evidence of the climatic aspects and measure them.

Organisational climate may affect quality of service and organisational commitment, and "general organisational climate can influence perception of safety climate, and these influence safety performance through their effects on knowledge and motivation" [35]. For this reason it could be important for management to pay attention to climate to ensure safety and quality of healthcare. The principle factor we found agrees with what Alpander stated about hospital employee motivation 20 years ago, "recognition is the primary motivating factor" [36].

## Competing interests

The author(s) declare that they have no competing interests.

## Authors' contributions

UW and RC conceived the ICONAS study within the regional CQI project, MB coordinated the design of the instrument, RC contributed to conception of the instrument as an organizational tool for the region's healthcare facilities, MB and UW designed the study, MB coordinated data collection and performed the statistical analysis, MB and UW drafted the manuscript, AN contributed to the interpretation of data, AN and RC revised the manuscript critically for important intellectual content, MB is the ICONAS project coordinator. All authors read and approved the final manuscript.

## Pre-publication history

The pre-publication history for this paper can be accessed here:


